# Recognition of Emotions Conveyed by Touch Through Force-Sensitive Screens: Observational Study of Humans and Machine Learning Techniques

**DOI:** 10.2196/10104

**Published:** 2018-08-30

**Authors:** Alicia Heraz, Manfred Clynes

**Affiliations:** ^1^ The Brain Mining Lab Montréal, QC Canada; ^2^ Institute of Amity and Emotion Research Nyack, NY United States

**Keywords:** emotional artificial intelligence, human-computer interaction, smartphone, force-sensitive screens, mental health, positive computing, artificial intelligence, emotions, emotional intelligence

## Abstract

**Background:**

Emotions affect our mental health: they influence our perception, alter our physical strength, and interfere with our reason. Emotions modulate our face, voice, and movements. When emotions are expressed through the voice or face, they are difficult to measure because cameras and microphones are not often used in real life in the same laboratory conditions where emotion detection algorithms perform well. With the increasing use of smartphones, the fact that we touch our phones, on average, thousands of times a day, and that emotions modulate our movements, we have an opportunity to explore emotional patterns in passive expressive touches and detect emotions, enabling us to empower smartphone apps with emotional intelligence.

**Objective:**

In this study, we asked 2 questions. (1) As emotions modulate our finger movements, will humans be able to recognize emotions by only looking at passive expressive touches? (2) Can we teach machines how to accurately recognize emotions from passive expressive touches?

**Methods:**

We were interested in 8 emotions: anger, awe, desire, fear, hate, grief, laughter, love (and no emotion). We conducted 2 experiments with 2 groups of participants: good imagers and emotionally aware participants formed group A, with the remainder forming group B. In the first experiment, we video recorded, for a few seconds, the expressive touches of group A, and we asked group B to guess the emotion of every expressive touch. In the second experiment, we trained group A to express every emotion on a force-sensitive smartphone. We then collected hundreds of thousands of their touches, and applied feature selection and machine learning techniques to detect emotions from the coordinates of participant’ finger touches, amount of force, and skin area, all as functions of time.

**Results:**

We recruited 117 volunteers: 15 were good imagers and emotionally aware (group A); the other 102 participants formed group B. In the first experiment, group B was able to successfully recognize all emotions (and no emotion) with a high 83.8% (769/918) accuracy: 49.0% (50/102) of them were 100% (450/450) correct and 25.5% (26/102) were 77.8% (182/234) correct. In the second experiment, we achieved a high 91.11% (2110/2316) classification accuracy in detecting all emotions (and no emotion) from 9 spatiotemporal features of group A touches.

**Conclusions:**

Emotions modulate our touches on force-sensitive screens, and humans have a natural ability to recognize other people’s emotions by watching prerecorded videos of their expressive touches. Machines can learn the same emotion recognition ability and do better than humans if they are allowed to continue learning on new data. It is possible to enable force-sensitive screens to recognize users’ emotions and share this emotional insight with users, increasing users’ emotional awareness and allowing researchers to design better technologies for well-being.

## Introduction

### Background

Emotions are distinct natural entities that involve the mind and the body. They modulate our physiology by increasing or decreasing variables such as heart rate, respiration, and body temperature, and our psychology by altering perception, beliefs, and virtual images [1,2]. Emotions seek expression, and they use one or many motor outputs (sequentially or simultaneously) to fulfill their need to be expressed: face, voice, arms, and legs are used in a combination of one or many outputs to express emotions depending on the context, the physical condition of the body, the available means of communication, and the choice of the expresser [1].

Touch is a profound form of communicating emotions, and many researchers have studied its use in and impact on health and well-being [1,3-9]. A few minutes of daily touches not only enhance growth and weight gain in children, but also lead to emotional, physical, and cognitive improvements in adults [6,7]. Touch releases hormones and neuropeptides, and stimulates our bodies to react in very specific ways: the levels of blood pressure, heart rate, and cortisol change, and the hippocampus area of the brain is activated for memory [3]. Humans can easily communicate and sense emotions conveyed through touch [8,9]; babies respond well to touch [4] and loving touches are critical to the health of premature infants [5].

We use touch to communicate with many devices in our daily lives. As machine interfaces are engaging users more frequently and tend to mimic human-human interactions to facilitate natural communications, it is becoming key to develop new algorithms for the new interactive and sensitive touch screens to capture and recognize users’ emotions and increase the level of emotional intelligence of both users and their devices.

Emotional intelligence, or our ability to recognize and regulate our own emotions and those of others, is key to communicating well. Recognizing emotions when they are expressed and regulating them in ourselves and others helps us achieve effective communications and maintain good mental health [10-12].

Emotional artificial intelligence, or a machine’s ability to express emotions and recognize and regulate users’ emotions, has also become a key capability of an intelligent machine enabling effective interactions with its users [13]. Various methods are being used and technologies are being built to detect users’ emotions as they interact passively or actively with machines. The most commonly used techniques for emotion recognition are one or a combination of many of the following approaches: text, facial expressions, voice tones, biosensors and body movements, and gestures.

Many technologies have implemented facial coding and voice analysis theories to recognize emotions, but none have explored the touch theory [1]. Software technologies analyze the face, tone of voice, and textual natural language to recognize emotions [14]. It is difficult to fulfill the requirements of these technologies and measure emotions accurately in real-life situations. It is essential to find and add other more accessible ways to measure emotions: that is, mobile phone use and touch screen behavior.

Emotion recognition techniques using text process words and sentences in a particular language. The most common techniques process natural language and extract emotions and sentiments from writings and conversations found in books, blogs, chat rooms, and social media platforms [15]. One of the biggest challenges of this method is to recognize emotions in the context of the text. An emotion can be expressed without using the word that denotes it or any of its synonyms. New words and expressions can be created, or words from other languages or sarcasm can be used to express emotions, which makes the task of recognizing emotions very difficult [16].

Usually, facial emotion recognition techniques segment images of the face into specific regions and analyze their movement. Regions of interest include cheek, chin, wrinkles, eyes, eyebrows, and mouth [17]. Different classification techniques are then applied to recognize emotions [18]. New studies have claimed that faces alone do not universally communicate emotions, and that conceptual knowledge supported by language is necessary to distinguish and recognize emotions [19,20]. In addition, a static image does not convey the information related to the dynamic form of the expression to accurately recognize emotions.

Speech emotion recognition techniques analyze the tone of voice and other speech features to detect emotions and their dimensions. Various methods and interfaces have been designed to extract various features from speech signals, and they are usually adapted to a particular language. The tone of speech varies with different cultures. A person from a certain area, talking in a normal tone, might sound angry to someone from another culture due to differences in normal speed and volume between the two cultures. Someone talking in a low and slow tone might appear as sad for some and polite for others. Additionally, speaking in real-life conditions is corrupted with various noises. This makes it hard for a machine to isolate a particular voice and recognize emotions [21].

Emotion recognition using biosensors monitors the physiological variables of the autonomic nervous system (ANS) that are affected by emotions. Biosensors can be invasive or noninvasive and collect variables in the ANS including heart rate, skin conductance, heart rhythm, blood volume, and temperature to recognize emotions through changes in their patterns. Spatial and temporal analysis of the brain’s activity are two other techniques that are used to recognize emotion [22]. Functional magnetic resonance imaging focusses on identifying the regions of the brain involved in expressing emotions, and electroencephalography monitors the electrical activity of the brain to recognize emotions. Different clustering and evaluating techniques are then used on these physiological changes to detect emotions. But consistent and universal patterns in the ANS in relation to emotions have not been found, and many technical challenges are still to be solved [12,23,24].

Body movements and finger gestures are other ways of expressing emotions. Features such as amplitude, speed, fluidity, shape of movements, and motion direction are being extracted from expressive children and adults, and various methods and techniques have been applied to recognize emotions [25-27]. A body action and posture coding system has been developed recently to enhance the understanding of the role of body movements in expressing emotions [28]. But emotion recognition based on body movements and gestures is the least popular way of evaluating emotions. Tracking body movements and gestures in 3 dimensions is difficult and requires many sensors, which is one of the major drawbacks of this method.

Multimodal approaches have also been used to recognize emotions. By analyzing two or many measures from the face, voice, text, or ANS, these approaches provide better accuracy than do individual modalities but are complex and not easy to replicate or scale [29-31].

In the last few years, researchers have started to explore correlations between emotions, features from smartphones, and gestures [28,32]. Analyzing data from a smartphone’s accelerometer predicted the emotional dimension of arousal with an accuracy of 75% [33]. Analyzing the length, time, velocity, and pressure of finger strokes on a smartphone predicted the emotional dimension of valence with 84.9% accuracy [34]. Analyzing features extracted from textual contents and user typing predicted anger, disgust, happiness, sadness, neutrality, surprise, and fear with 72% accuracy [35].

The worldwide number of mobile phone users is expected to pass the 5 billion mark by 2019 [36]. Most of the mobile market growth can be attributed to the increasing popularity of smartphones. Users touch their smartphones thousands of times a day [37]: they play games, purchase products, and interact with other users in chat rooms and social media platforms. Users spend 54 minutes to 3.8 hours per day on their smartphone. Among 16- to 24-year-olds, 94% possess a smartphone and spend up to 4 hours per day on it. They open an app every 15 minutes because they feel the urge to do so: it is difficult for most of them to reduce the time spent on a smartphone or control the frequency of its use [38]. Addictive behaviors are dictated by uncontrolled emotions, where reasoning and logical thinking is not applied [2,39]. The World Health Organization classifies addictive behaviors for technology as a mental health problem [40].

It is thus essential to develop technologies for emotional awareness and help users establish a healthy communication between reason and emotions to lower addictive behaviors and suffering, improve decision making, and increase well-being [41].

### Objective

This study explored the power of touch and its potential to convey distinct emotions when it is used as a means to communicate with apps and distant users via force-sensitive smartphones. Our ultimate goal is to increase users’ emotional awareness by powering smartphone apps with touchscreen emotional intelligence. We hope to open new opportunities for designers to create new interactive emotional experiences, provide emotional inputs for developers to enrich their apps, and offer insight for researchers to better understand emotions in the context of human-computer interactions.

We conducted 2 experiments in this study to recognize anger, awe, desire, fear, hate, grief, laughter, love, and no emotion. Experiment 1 examined whether humans are able to recognize the emotions by looking at passive expressive touches. Experiment 2 collected features of expressive touches of emotionally aware participants with good imagery ability and used machine learning techniques to predict emotions.

## Methods

### Emotions

Emotions are complex entities that often cannot be defined with just one single word. Naming an emotion is labelling the qualities of its psychophysiological manifestation, and these qualities are not precisely known. Naming by language the experience of anger is not a guarantee of the existence of a simple and clear psychophysiological pattern. Anger is not a simple entity, and complex and mixed patterns may have simple names. Sometimes the naming is, to a degree, confused and confusing. Some emotions remain nameless.

In this study, we were interested in what we define as *biological emotions* or nonverbal emotions for which language is not required when they are communicated. We listed 8 emotions (and no emotion) that we considered to be biological, and we hypothesized that they are easily recognizable by a perceiver if expressed authentically: anger, awe, desire, fear, grief, hate, laughter, and love, as well as no emotion. The main word we coined for each emotion is approximate and not unique. Translating our words to other languages and explaining them to our participants required that we define every individual word by describing how people react and what they say and do when they are under the influence of that emotion. Table 1 describes our 8 biological emotions.

In addition to these descriptions, we added a set of images and videos showing people’s faces, speech, and body movements for every emotion. We collected this multimedia content from the internet. We were inspired by the International Affective Picture System [42] and the affective videos of the Laboratoire d’Informatique en Image et Systèmes d’information Annotated Creative Commons Emotional Database [43], and standardized in terms of its audio and visual characteristics (brightness, loudness, distance, color, and size).

### Participant Recruitment

We recruited and screened volunteers and smartphone users before assigning them into 2 groups to participate in 2 experiments. We contacted a local volunteer center and posted advertisements on websites asking people above 16 years old who were interested in emotions and technology to participate in our study for free.

**Table 1:** Labels, descriptions, and synonyms of the 8 biological emotions (and no emotion).

Label	Description	Synonyms
Anger	When you are angry, you boil, react, object, yell, or swear. You say words or expressions like “fuck,” “shit,” “no,” “stop,” or other synonyms silently in your head or loudly in your own language, usually your native language.	Frustration Rage Fury
Awe	When you are in awe, you freeze or slow in contemplation. You disconnect from distractions and get absorbed by the object of your awe. You are speechless and cannot link what you discover with what you already know.	Interest Discovery Contemplation
Desire	When you desire, you want, crave, need, and starve for. You say words or interjections like “yummy,” “come,” “tasty,” “want you,” or other synonyms silently in your head or loudly in your own language, usually your native language.	Need Lust Want
Fear	When you fear, you withdraw, hide, freeze, or tremble. You remain silent or say words or expressions like “no” or other synonyms silently in your head or loudly in your own language, usually your native language.	Scare Panic Terror
Grief	When you are in grief, you are very sad, and feel helpless and weak. You suffer and feel pain. You cry, moan, and whimper.	Agony Mourning Sadness
Hate	When you hate, you destroy, crush, and break. You say words or expressions like “perish” or “die” in your head or loudly in your own language, usually your native language.	Detestation Loathing Vengefulness
Laughter	When you laugh, your breath and voice are chopped and your eyes twinkle and tear. You repeat “Ha ha” or other sounds while you move in the same rate as you laugh and emit sounds.	Chuckle Giggle Excitement
Love	When you love, you care, protect, comfort, and maintain the state of the loved object. You smile, remain silent, or say words or expressions like “dear,” “cute,” or “sweet” in your head or loudly in your own language, usually your native language.	Affection Delight Joy
No emotion	When you are not under the influence of an emotion, you reason with ease. Counting from 1 to 10 while seeing or visualizing the numbers in your head is an example of a very simple and unemotional state.	Reasoning Thinking Counting

### Grouping Procedure

We invited all volunteers to complete 2 tests: the Levels of Emotional Awareness Scale (LEAS) to assess their emotional awareness [44]; and the Questionnaire Upon Mental Imagery (QMI) to assess their imagery ability [45,46]. For the purpose of our study, and the requirement to express pure and authentic emotions in both experiments, we needed all participants in group A to be not only emotionally aware, but also able to physiologically react to imaginary emotional situations. Only 20 participants among the 117 volunteers accepted to take the tests and apply to be part of group A.

The LEAS is an open-ended test in which we assessed the ability of volunteers to use emotion words in various situations to describe their feelings and the feelings of others [44]. A total of 20 volunteers answered 20 questions describing 20 emotionally evocative situations, and we hand-scored their responses. The scoring was as follow: 0 was given to nonemotional responses or when a response described thought instead of feeling; 1 was given when participants described physical awareness (eg, “I feel tired”); 2 was given when a response described an undifferentiated emotion (eg, “I feel bad”) or when the response described an action (eg, “I feel like I’m going to punch him in the face”); 3 was given when feelings were described using discrete emotions (we have a glossary of more than 600 discrete emotions collected from prior studies); 4 was given when many different discrete emotions were used to describe mixed or complex feelings; and 5 was given when participants described and differentiated their own feeling from someone else’s feelings using discrete emotions [47].

A higher score in the LEAS correlates positively with empathy, understanding of others, and openness to experience [48], as well as the ability to recognize emotions [49,50].

The QMI is a 600-item measure of mental imagery ability for 7 sensory modalities (visual, auditory, cutaneous, kinesthetic, gustatory, olfactory, and organic). In our study, we focused on the visual, cutaneous, and kinesthetic sensors only (the scoring of each of the 7 modalities is independent, and the emotion induction protocol requires participants to focus on 3 senses). We thus asked our volunteers 80 selected questions similar to the original questions of the test to indicate how clearly they could imagine a series of situations (eg, the form and movement of an assassin approaching the bed, touching silk, running fast to catch a car). Scoring was on a 7-point vividness rating scale varying from 1 (“Perfectly clear and as vivid as the actual experience”) to 7 (“I think about it but I cannot imagine it”), with a total score ranging from 80 to 560. Higher total scores indicate weaker imagery ability. Physiological activity in response to emotional imagery varies as a function of imagery ability. This means that good imagers show greater emotion-specific physiological activity than poor imagers [51].

We conducted experiments 1 and 2 in the laboratory on the same day for group A. Group B participated in experiment 1 remotely. We asked group A participants to follow some instructions (see below) 1 day before the day of the experiments. On the day of the experiments, they participated in an interview session and a training session before experiments 1 and 2 [52].

### Instructions for the Day Before

We asked group A to rest, sleep, and eat well but not too much, because emotions are subject to physical and psychological states and can be difficult to induce under conditions of stress, fatigue, lack of sleep, hunger, or heavy meals [1]. For the needs of the training session, we asked them to come, if possible, with a voluntary partner with whom they were able to be emotionally intimate. Alternatively, we set up a time when 2 single group A participants could come together in the same time and partner each other during the training session. We asked all of them to bring their smartphone.

### Interview Session

We excluded group A participants if they had a history of any of the following: current alcohol and drug abuse or dependence, neurological disease or trauma, and other medical or psychiatric complications. To share our definition of emotions clearly with everyone in group A, we gave them the list of emotions described in Table 1, and they viewed our collected multimedia content for each emotion. We encouraged them to ask questions and translate the words we gave as examples to their own words and language. All group A participants signed a consent form where a strict ethics code was applied, including their right to ask questions, withdraw from the experiment, keep their personal information private, and understand the scope and the purpose of the study.

### Smartphone Test

Our protocol in experiment 2 required a minimal granularity in the measurement of finger pressure and area to collect enough change in data for our machine learning algorithms. To test whether a smartphone is sensitive enough, we designed an app that can be set up on any smartphone to check our requirements. After installing and launching the app on their smartphone, we asked participants to press on their screen as hard as possible for 5 seconds. We required a minimum granularity level of 10 for both area and force, a minimum pixel density of 100 pixels per inch, and a maximum temporal resolution of 1 millisecond. We used the device model Motorola XT1023 (Motorola Mobility LLC, Libertyville, IL, USA) with all of those who did not have a sensitive enough smartphone.

### Training Session

The purpose of this session was to train participants from group A to express each emotion as precisely as possible by using only their finger and touching the palm of the partner to communicate each emotion. We called group A participants “expressers” and their partners “perceivers.” We encouraged but did not oblige expressers to use their middle finger during the expression because it is the first finger to reach the target of touch and the least cumbersome to use.

We asked the expressers to read the description of each emotion (see Table 1) and use our inductive material (images and videos) to stimulate their imagination and remember an emotion-provoking life situation. We asked them to describe the situation as if they were actively involved and emotional. They were encouraged to close their eyes and imagine the specific situation so as to experience it more accurately. A blank paper was provided, and participants were required to write down a description of the situation, their thoughts, and their words for each emotion. They were encouraged to use their own language. Textbox 1 lists the instructions given to the expressers.

We gave the perceivers, in a separate room, the list of emotions (see Table 1) and asked them to memorize them. They were informed that they were to be blindfolded and seated on a chair with their palm resting on their knee, and that an expresser would try to communicate an emotion from the list by only touching the perceiver’s palm with their finger. We asked the perceivers to try to guess the emotion conveyed by the finger and respond with 1 of the following 3 options: 1: “I don’t know;” 2: “I hesitate between...;” and 3: “I know; it is...”

**Figure 1:**
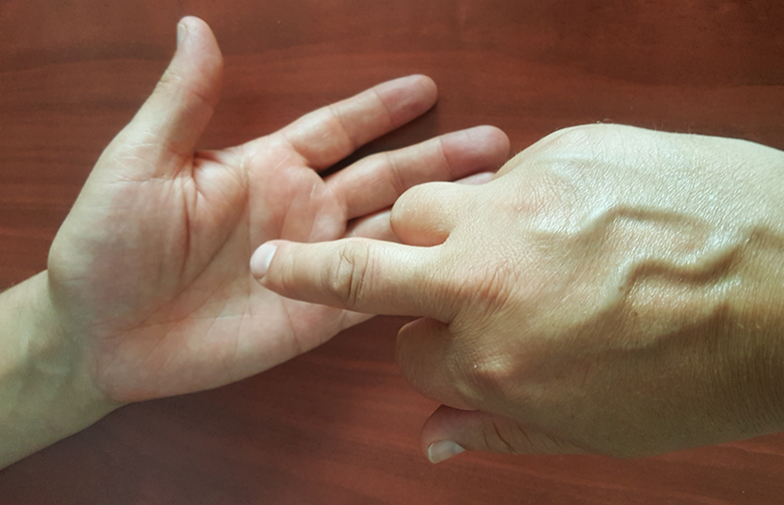
Group A participant expressing emotions in the palm of their partner.

We asked expressers to express each emotion once or many times until the perceiver guessed their emotion correctly. They were free to move to another emotion before coming back to an emotion they had already tried to express without being successful and try again until they succeeded. Expressers were given a paper on which the emotions were listed and were also asked to put 1 or 0 at every trial (1: their partner successfully recognized the emotion; 0: their partner was confused or not able to recognize the emotion). Figure 1 shows the emotional expression of participants on the palms of their partners.

We asked expressers who completed the training session to participate in experiments 1 and 2, but only to express the emotions they were able to communicate successfully to their perceivers.

### Experiment 1

The objective of this experiment was to answer the following question: if emotions modulate our touches [1,8,9], will humans be able to recognize emotions by only looking at an expressive touch?

**Instructions to induce and express emotions.**

Choose an emotion from the list. Remember the related scene that you described as vividly as possible. Imagine as if it were really happening to you. Communicate your emotion by touch as precisely as possible.

We asked participants from group A to express the emotions that their partners were able to recognize during the training session against a transparent glass under which we video recorded their touches for 30 seconds. Each session started with neutral (nonemotional) touches.

**Figure 2:**
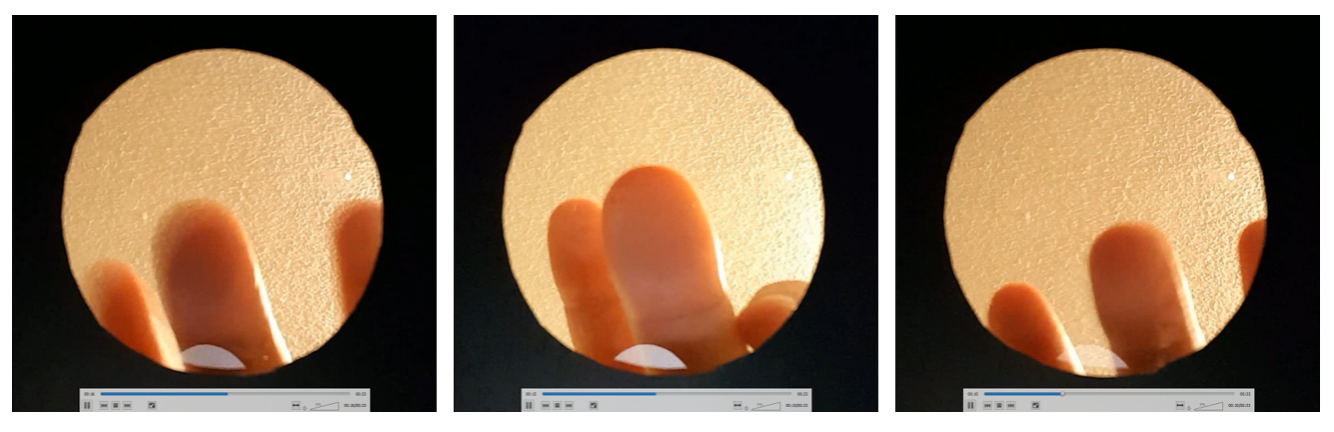
Frames from the expression through touch of fear (left), grief (middle), and laughter (right).

We asked participants to express each emotion with one or many successive touches. They were encouraged but not obliged to use their middle finger. Figure 2 shows 3 frames from 3 different videos in which a participant expressed fear, grief, and laughter, respectively (left to right).

We then showed the videos online to group B and asked them to guess the emotion expressed by the finger by classifying the expression as 1 of the 8 emotions (and no emotion) described in Table 1.

### Experiment 2

The objective of this experiment was to answer the following question: can we teach machines how to accurately recognize users’ emotions from their touches on force-sensitive screens?

We asked group A participants to express the emotions that their partners were able to recognize during the training session against an app that we installed on a smartphone. They were encouraged to use their middle finger because it is the least cumbersome finger and the first to reach and touch the screen, but they were allowed to use any other finger if it was easiest for them to use.

**Figure 3:**
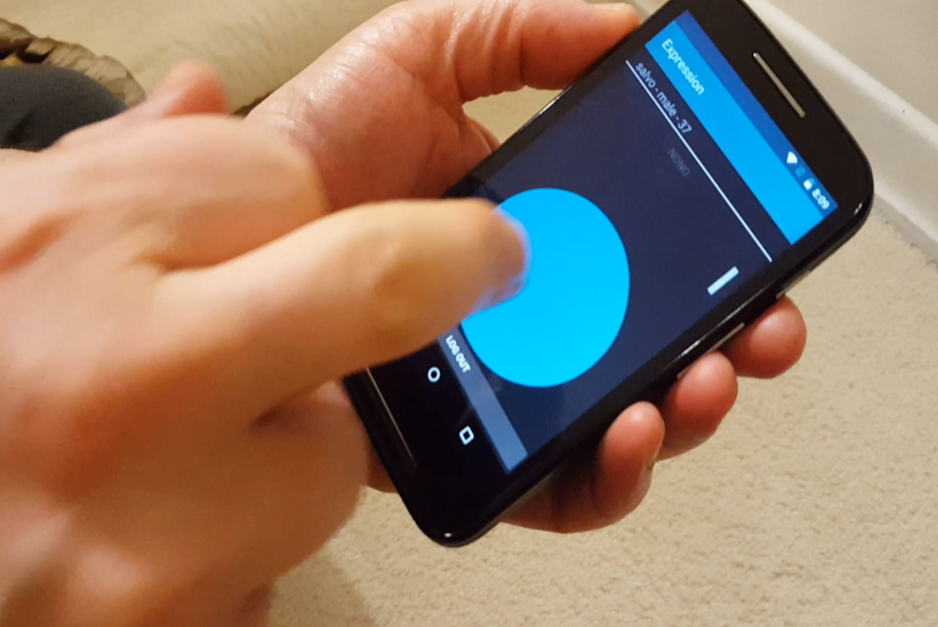
Emotional expressions on the mobile app.

Figure 3 shows the app’s interface and a participant expressing an emotion. Participants followed the same instructions as those in the training session (see Textbox 1).

Each session started with neutral (nonemotional) touches. We asked participants to express each emotion with one or many successive touches. They were encouraged to express each emotion at least 10 times, successively or not. After each emotional expression, participants took a 5-minute break to relax and resume a neutral state, thus preventing potential carryover effects of the previous emotional experience. During this period, we asked participants to rate on a single 5-level scale the purity of their emotional expression and the extent to which their finger expression conveyed the emotion. The rating varied from 0 to 5, where 0 was “not expressed” and 5 was “expressed well.” This allowed us to assess the accuracy of the induction procedure, as well as the subjective state of the participant after the emotional experience and expression.

#### Precision, Calibration, and Normalization

We recorded the coordinates (x_t_,y_t_) of finger touches, their amount of force (F_t_), and skin area (S_t_), all as functions of time with a resolution of 1 millisecond. Knowing the screen density of each device, we converted (x_t_,y_t_) from pixels to millimeters with a precision of a hundredth of a millimeter. We calibrated F_t_ and S_t_, knowing the maximum amount of force and skin area a participant could apply on a device, and then normalized it on a scale of 0 to 1 with a precision of 2 digits after the decimal, because the maximum values F_t_ and S_t_ differ not only between devices but also between participants. Some devices are more sensitive than others, and the maximum values of F_t_ and S_t_ are not always 1. Participants had different finger sizes, and large fingers record larger areas.

#### Data Filtering, Touches, and Expressions

Among the data we collected, and based on the subjective rating, we retained only the expressions that were rated 3 or more on the scale of purity. We grouped successive touches with a delay of less than 1000 milliseconds and considered them to be part of one single emotional expression. Expressions comprised one or many touches depending on the emotion, its intensity, and the need to express it.

**Table 2:** Feature dependency test using paired *t* test.

Pair	Mean (SD)	SE	95% CI
1	5.139 (69.361)	1.441	2.313 to 7.966
2	175.118 (1000.759)	20.795	134.339 to 215.897
3	0.756 (1.520)	0.032	0.695 to 0.818
4	–284.59 (3446.514)	71.616	–425.03 to –144.15

#### Feature Extraction

To set up an initial set of relevant features and observe the expression of each emotion in time, we designed a visualization tool using Python.

#### Dimension Reduction

Based on our intuitive assumptions and observations and the experimental context, we calculated for every expression an initial set of the following 17 spatiotemporal features: the coordinates (x1, y1, x2, y2) of the beginning and end of an expression; the distance, angle, duration, velocity, and acceleration of the expression; the total number of touches and the mean duration of touches of an expression; and the mean, maximum, and velocity of both force and size of an expression. Further statistical analysis and feature selection techniques including principal component analysis [53] allowed us to reduce the number of features to 9 independent features. We retained the first 9 eigenvectors because they captured 95% of the total variance in the original data. The distribution of variance among the 9 components was 20.1% (roughly corresponds to the duration), 18.2% (the amount of force), 12.7% (the number of touches), 11.3% (the spatial extent on the x,y axis), 10.4% (the angle), 8.2% (the distance), 5.1% (the size), 4.6% (the velocity), and 4.4% (the acceleration).

We used the parametric paired *t* test to measure the degree of variance and dependency between the normally distributed features. Table 2 displays the comparison results.

We see that the mean differences 5.139 (in pair 1), 175.118 (in pair 2), 0.756 (in pair 3), and –284.59 (in pair 4) between features are not equal to zero. With 95% confidence (*P*=.05), we could conclude that there was a significant statistical score to indicate that our selected features were independent.

## Results

### Participants

We recruited and screened 117 volunteers and smartphone users between the ages of 16 and 63 years (47 male and 70 female participants). Their mean age was 32.5 (SD 13.9) years.

Only 15 participants were rated as good imagers and highly emotionally aware enough to be part of group A (15/117, 12.8%; 7 male and 8 female participants). They were all right-handed and scored above 70 (very much above average) in the LEAS test and below 107 (above average) in the QMI.

**Figure 4:**
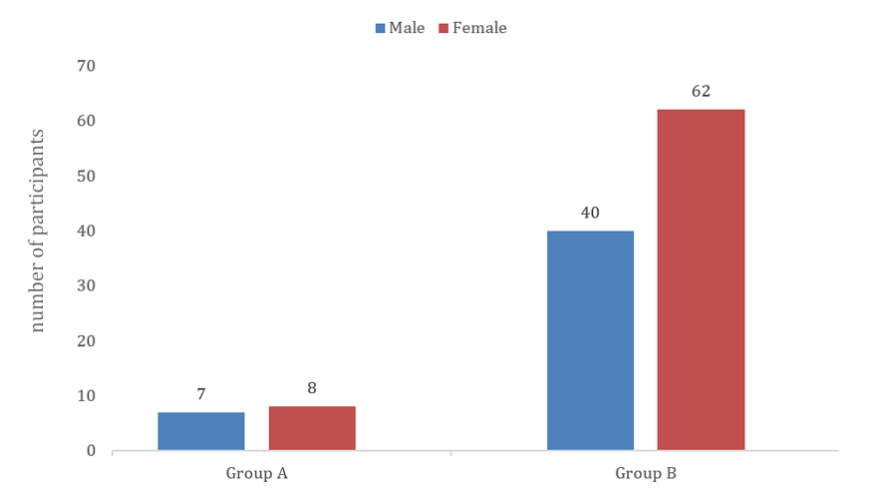
Group and sex distributions of the participants.

The other 5 participants were either not enough emotionally aware or not good enough imagers, or both. We assigned them to group B, which comprised 102 volunteers (40 male and 62 female participants), and we applied no additional tests or requirements to them. Both groups participated in experiment 1, and only group A participated in experiment 2. Figure 4 shows the distribution of the participants by group and sex.

### Smartphone Sensitivity Tests

We tested 11 different smartphone devices: 63% (7/11) were eligible to be used in experiment 2 (see Table 3).

### Experiment 1: Human Recognition for Emotions

We collected 102 responses for each emotion. The results proved to be highly successful and significant, with all 8 emotions (and no emotion) correctly recognized with great accuracy with only 1 attempt. Errors of recognition were mainly choosing hate for anger (7/918, 0.8%) and vice versa, or choosing awe for no emotion (11/918, 1.2%) and vice versa. A total of 12 of the 918 participants (1.3%) confused hate with laughter.

**Table 3:** Sensitivity tests on participants’ smartphones.

Device model	Screen density (pixels/inch)	Force granularity	Area granularity	Sensitive enough?
Apple iPhone 6S	326	121	95	Yes
Huawei G610U20	220	120	32	Yes
Huawei Y635TL00	196	1	1	No
LGE-D802	424	30	12	Yes
Motorola XT1023	256	52	16	Yes
OnePlus A2003	401	1	5	No
Samsung G920A	576	1	41	No
Samsung I8530	233	15	18	Yes
Sony Xperia XZ	424	35	21	Yes
Xiaomi RNote3	403	48	14	Yes
Xiaomi Redmi4	294	1	1	No

**Figure 5:**
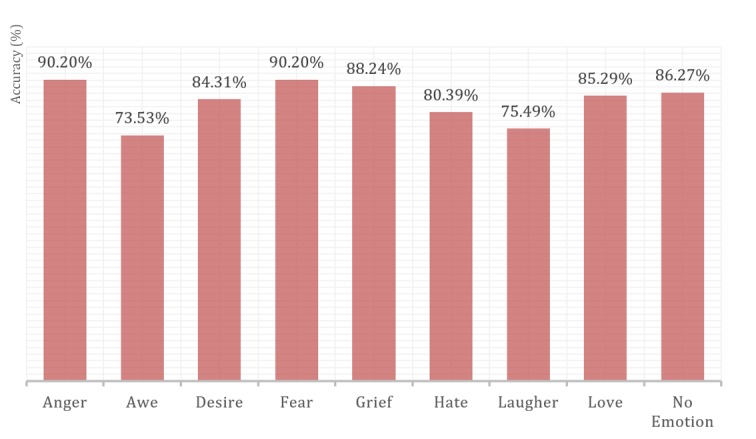
Human recognition of emotions in force-expressive touches.

However, 49.0% (50/102) of the participants were 100% (450/450) accurate in recognizing all 8 emotions (and no emotion) and 25.5% (26/102) were 77.8% (182/234) accurate. Male and female participants did equally well. Figure 5 shows the results obtained for each emotion and Table 4 details the classification results for each emotion. This experiment confirmed that emotions modulate our fingers as we force-touch a sensitive surface, as well as that humans have a high ability to recognize emotions from the pattern of the emotion in the expressive force-touch.

We can see distinct patterns between emotions in terms of their beginning and end, as well as the shape, speed, acceleration, and space occupation of their respective expressions. Figure 7 shows more details in the variation of the amount of force and skin area for each emotion. The horizontal axis is the time in milliseconds; the vertical axis represents the variation of force from 0 to 1, and the dot size on the curves correlates with the variation of the skin area over time.

### Data Visualization

**Figure 6:**
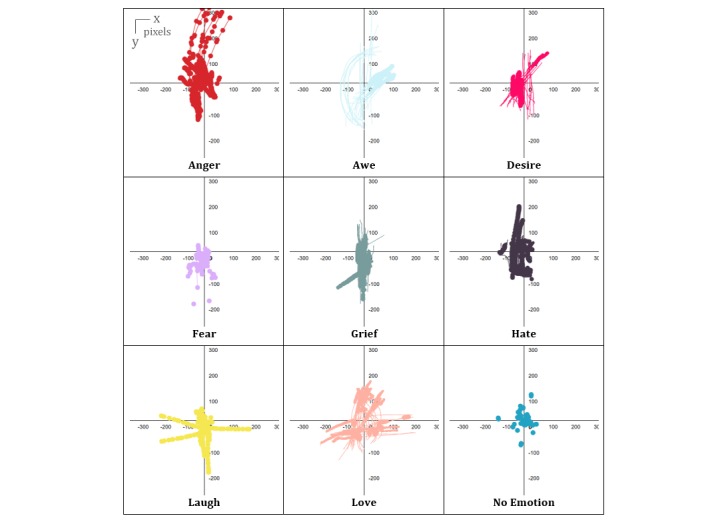
Patterns of emotional expressions on the (x,y) axis.

Figure 6 shows a single frame taken from participants’ finger expressions of each emotion at a random instant t on the (x,y) axis.

Emotional expressions have distinct amounts of force and skin area. Hate is characterized by a very high amount of force and fear is very fast and short.

**Table 4:** Classification results of group B for each video.

Emotion expressed in the video	Participants’ classification								
	Anger	Awe	Desire	Fear	Grief	Hate	Laughter	Love	No emotion
Anger	92	2	0	0	1	5	2	0	0
Awe	1	75	3	2	5	4	6	2	4
Desire	1	5	86	0	1	2	2	4	1
Fear	1	2	0	92	0	1	4	1	1
Grief	1	4	2	0	90	1	2	2	0
Hate	2	2	1	2	2	82	9	2	0
Laughter	4	2	2	2	2	3	77	4	6
Love	0	3	4	2	1	1	2	87	2
No emotion	0	7	3	2	0	1	1	0	88

### Experiment 2: Machine Learning Classification

Table 5 shows the results of overall subjective ratings of group A for each emotion.

All participants expressed anger and no emotion very well (486/486, 100% of expressions were rated 5). Grief and laughter were the most challenging (20% of expressions: 25/126 for grief and 46/232 for laughter were rated below 3).

Our dataset comprised 2316 instances (or emotional expressions). Figure 8 shows the distribution of instances among emotions. Anger was the most expressed emotion with 486 expressions, and grief was the least expressed with 101 instances.

The number of touches per emotional expression varied between 1 and 25 (mean 1.94, SD 2.88); 83.46% (1933/2316) of expressions had an average of 1.38 touches and 3.45% (80/2316) of expressions had a maximum of 2.5 touches (see Figure 9). We saw more touches in laughter and anger than in the other emotions.

**Figure 7:**
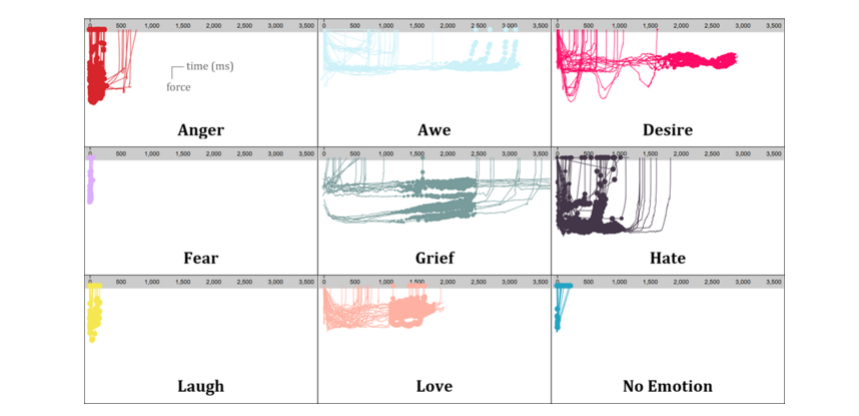
Force and skin variation of emotional expressions in time. The horizontal axis is the time in milliseconds; the vertical axis represents the variation of force from 0 to 1, and the dot size on the curves correlates with the variation of the skin area over time.

We designed an experimental framework in which we tested various machine learning techniques in supervised learning, including naive Bayes, nearest neighbor, neural networks, meta, and decision tree classifiers. We obtained the best classification results in random committee and 2 decision tree algorithms: random tree and random forest.

Random committee is a type of meta algorithm that takes classifiers and converts them into more powerful learners. Random committee builds an ensemble of base classifiers and averages their predictions [54]. Each one is based on the same data but uses a different random number seed. Decision trees are treelike structures; they start from root attributes and end with leaf nodes. Decision tree algorithms describe the relationships among attributes and the relative importance of attributes. Random tree chooses a test based on a given number of random features at each node, performing no pruning. Random forest constructs random forests by bagging ensembles of random trees [55]. Table 6 shows the classification results using the 10-fold cross-validation test option.

The percentage of correctly classified instances was very high, and varying between 86.14% (1995/2316) and 91.11% (2110/2316). Kappa statistics is a chance-corrected measure of agreement between the classifications and the true classes. It is calculated by taking the agreement expected by chance away from the observed agreement and dividing by the maximum possible agreement. Kappa being higher than .81 demonstrates an almost perfect agreement for all the classifiers. Random forest produced the best results. Table 7 shows its detailed accuracy per emotion and Table 8 shows the confusion matrix.

The rate of true positives (or recall) varied from .75 for awe to 1.00 for fear. Most instances were correctly classified. The rate of false positive was insignificant and was highest in love, with only .03 instances falsely classified. The proportion of instances that were truly of a class divided by the total instances classified as that class (precision) varied from .82 to 1.00. A combined measure for precision and recall calculated as 2 × precision × recall / (precision + recall) is presented as the F measure. The area under the receiver operating characteristic curve approach 1.00 for all classes (>.98), which demonstrates the optimality of our model.

The confusion matrix in Table 8 shows the raw numbers, with anger, are, desire, fear, hate, grief, laughter, love, and no emotion being the class labels.

To test the performance of the classifier, we trained it on 15 subsets where we excluded 1 different participant in each run to be tested on the sample of the excluded participant (leave-one-run-out cross-validation and leave-one-sample-out cross-validation). The average performance of the classifier was 86.36%, kappa=.84: a drop of 4.6% compared with the result obtained with 10-fold cross-validation.

### Human Versus Machines

In recognizing our emotions from finger force-touches, our algorithm did better than group B participants. Figure 10 shows a comparison of accuracy between our algorithm and group B participants in recognizing emotions.

For group B participants, anger was the easiest recognizable emotion, while awe and laughter were the most difficult to guess. Our algorithms were best in detecting fear, with almost 100% accuracy.

**Table 5:** Group A subjective ratings (range 0-5) for each emotion in experiment 2, by proportion giving that rating.

Emotion and rating		Participants who chose the rating, n (%)
**Anger (n=486)**		
	5	486 (100)
**Awe (n=181)**		
	4	23 (12.7)
	5	158 (87.3)
**Desire (n=302)**		
	2	39 (12.9)
	3	60 (19.9)
	4	82 (27.2)
	5	121 (40.0)
**Fear (n=292)**		
	4	79 (27.1)
	5	213 (72.9)
**Grief (n=126)**		
	0	25 (19.8)
	4	25 (19.8)
	5	76 (60.4)
**Hate (n=303)**		
	4	61 (20.1)
	5	242 (79.9)
**Laughter (n=232)**		
	0	23 (9.9)
	1	23 (9.9)
	4	46 (19.8)
	5	140 (60.4)
**Love (n=288)**		
	4	95 (33.0)
	5	193 (67.0)
**No emotion (n=216)**		
	5	216 (100)

**Figure 8:**
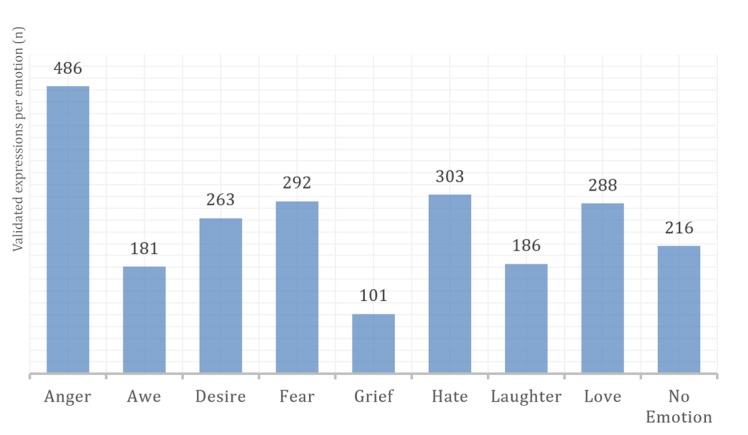
Overall number of expressions for each emotion.

**Figure 9:**
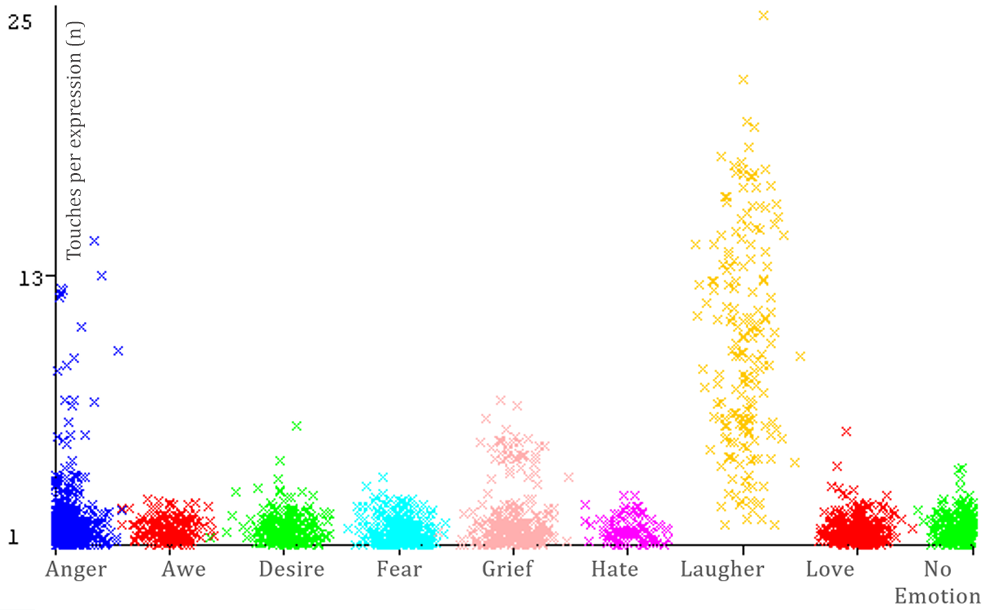
Number of touches per expression for each emotion.

**Table 6:** Best emotion classification results using the 10-fold cross-validation test option.

Algorithm	Correctly classified, n (%)	Kappa statistic	Mean absolute error	Root mean square error	Relative absolute error (%)	Root relative square error (%)
Random tree	1995 (86.14)	.84	.03	.18	15.90	56.40
Random committee	2082 (89.90)	.88	.03	.13	16.43	42.62
Random forest	2110 (91.11)	.90	.04	.13	19.54	40.77

**Table 7:** Detailed accuracy per class for the random forest classifier.

Class	True positive rate	False positive rate	Precision	Recall	F measure	Matthews correlation coefficient	Area under the receiver operating characteristic curve	Area under the precision-recall curve
Anger	.957	.013	.951	.957	.954	.942	.994	.981
Awe	.746	.014	.818	.746	.780	.764	.980	.814
Desire	.814	.016	.866	.814	.839	.820	.980	.911
Fear	1.000	.000	1.000	1.000	1.000	1.000	1.000	1.000
Hate	.904	.016	.893	.904	.898	.883	.991	.940
Grief	.871	.008	.838	.871	.854	.848	.996	.926
Laughter	.984	.001	.989	.984	.987	.985	1.000	.999
Love	.865	.030	.803	.865	.833	.809	.978	.886
No emotion	.972	.003	.972	.972	.972	.969	.999	.995
Weighted average	.911	.012	.911	.911	.911	.899	.991	.946

**Table 8:** Confusion matrix for the random forest classifier.

Class	Anger	Awe	Desire	Fear	Hate	Grief	Laughter	Love	No emotion
Anger	465	5	1	0	0	0	2	10	3
Awe	0	135	12	0	0	5	0	29	0
Desire	2	4	214	0	28	1	0	13	1
Fear	0	0	0	292	0	0	0	0	0
Hate	3	2	14	0	274	7	0	3	0
Grief	3	3	0	0	2	88	0	5	0
Laughter	3	0	0	0	0	0	183	0	0
Love	10	15	5	0	3	4	0	249	2
No emotion	3	1	1	0	0	0	0	1	210

**Figure 10:**
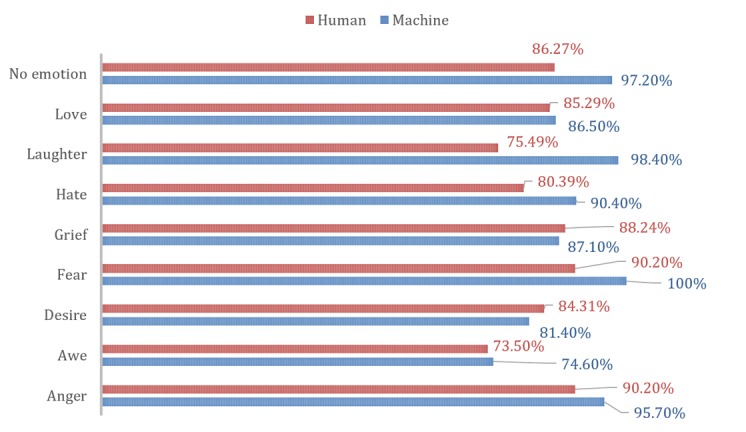
Human versus machine algorithm recognition of emotions.

## Discussion

### Principal Results

The results of the first experiment of this study demonstrated the ability of humans to recognize emotions when expressed through finger force-touch, and the results of the second experiment demonstrated clear finger force-touch patterning of emotions.

Our findings in experiment 1 indicated the ability of group B participants to recognize 8 emotions: anger, awe, desire, fear, grief, hate, laughter, love (and no emotion) as described in Table 1. Group B had a high accuracy of 83.8% (769/918) in recognizing the emotions of the emotionally expressive participants (group A) by only looking at the movement of their fingers when pressing a transparent glass to express emotions. Clynes [1] reported a similar ability of humans to recognize emotions by only looking at the movement in space of an arm expressing emotions. Clynes [1] and Hertenstein and colleagues [8,9] demonstrated the ability of humans to communicate and perceive distinct emotions via touch. Our finding in experiment 2 indicated higher accuracy for our algorithm, recognizing clear patterns of 8 emotions (and no emotion) with a 91.11% (2110/2316) classification accuracy. Following the training sessions, recording finger force-touch and skin area in the expression of each emotion and filtering the collected data based on subjective ratings revealed highly significant and accurate classification of group A’s emotions.

An interesting insight in our data was the correlation between the 8 emotions (and no emotion) and the spatiotemporal features: coordinates of the beginning and end of an expression, the distance, angle, duration, velocity, and acceleration of the expression, the total number of touches, the mean duration of touches of an expression, and the mean, maximum, and velocity of both force and size of an expression.

### Limitations

Using a strict participant selection process and a personalized emotion induction protocol with human validation and subjective rating allowed us to state that anger, awe, desire, fear, grief, hate, laughter, love, and no emotion produce specific response patterns in finger force-touch expression of emotions. However, there are several considerations (related to the selection process of group A participants) that limit generalization of our findings: group A participants (1) were highly emotionally aware, (2) had good imagery ability, (3) were used to smartphones, (4) had good touch dexterity on their smartphones, and (5) were willing and able to communicate emotions authentically using touch. Future replication of these findings is needed in participants who are poor imagers, less emotionally aware, and nonusers of smartphones.

### Comparison With Prior Work

According to cognitive-physiological network models, ideas, memories, and verbal and subjective descriptors are very important components to induce emotions. Thus, when we scrupulously described our emotions to our participants and then asked them to remember and personalize meaningful emotion-evoking scenes and use their finger as the motor output to communicate the emotion to another participant before communicating the expression to a smartphone, the finger force-touch alone discriminated between anger, awe, desire, fear, grief, hate, laughter, love, and no emotion in 91.11% (2110/2316) of the cases. This indicates that the 9 spatiotemporal features extracted from finger force-touch measures were part of the network that was activated when participants expressed their emotions. Our accuracy rates were higher than those reported in earlier similar studies where emotions or emotional dimensions were detected from force-touch, typing, and words or other sensors in the smartphone [28,32-35]. This may be related to clearer descriptions of our target emotions, the use of personalized multimedia material and imagery of real-life situations to induce emotions, and the use of another participant to validate the communication of the emotion via finger force-touch. Also, our higher accuracy may be due to the strict conditions in the selection process of participants for group A (they were all good imagers and highly emotionally aware).

### Conclusions

Emotions are unique and important entities with built-in windows across the mind-body barrier that need to be understood. They convey great power in the development and mental healing of the individual, of society, and even for the now self-conscious evolution of human beings.

In this study, we described a protocol and implemented methods that allowed us to validate the human ability to express and perceive distinct emotions, and a machine’s ability to recognize those emotions on force-sensitive smartphones. Much remains to be done to build more comprehensive, accurate, and scalable emotion detection algorithms in real-life contexts.

Touch is one of the most powerful of human senses, and we use it passively to communicate emotions. As we continue interacting with devices through touch, it is becoming essential to analyze these patterns and sense emotion. Enabling smartphone apps to capture, discern, and communicate the emotions of expressive touches will completely change the way users perceive and touch their devices and facilitate spontaneous emotional expressions. Human-computer-human interactions will get better, clearer, and emotionally intelligent.

## Abbreviations

ANS: autonomic nervous system
LEAS: Levels of Emotional Awareness Scale
QMI: Questionnaire Upon Mental Imagery
